# Treatment of Singultus by Sulphuric Acid

**Published:** 1850-10

**Authors:** 


*Treatment of Singultus by Sulphuric Acid.* By Dr. Schneider.—During a long practice, Dr. Schneider has met with many cases of hiccough, occurring in both sexes and at different ages, and persisting with such obstinacy as to give rise to great suffering and exhaustion. His sovereign remedy in such cases is one of the preparations of dilute sulphuric acid, which act with great promptitude. He refers also tc the testimony of Dr. Duncan, in favor of this substance contained in the 'Edinb. Med. Comment.,' and to that of Dr. Jacobsen, of Berlin.—('Casper's Wochenschrift.')—*Brit. and For. Review, Jan.* 1850, *p.* 274.

